# Modulation of the Neuroprotective and Anti-inflammatory Activities of the Flavonol Fisetin by the Transition Metals Iron and Copper

**DOI:** 10.3390/antiox9111113

**Published:** 2020-11-11

**Authors:** Pamela Maher

**Affiliations:** Cellular Neurobiology Laboratory, Salk Institute for Biological Studies, La Jolla, CA 92037, USA; pmaher@salk.edu

**Keywords:** oxidative stress, glutathione, ferroptosis, oxytosis, Nrf2, Alzheimer’s disease, sterubin

## Abstract

Alterations occur in the homeostasis of the transition metals iron (Fe^2+^) and copper (Cu^2+^) during aging and these are further amplified in neurodegenerative diseases, including Alzheimer’s disease (AD). These observations suggest that the most effective drug candidates for AD might be those that can reduce these alterations. The flavonoid fisetin has both neuroprotective and anti-inflammatory activity both in vitro and in vivo and can bind both iron and copper suggesting that its chelating activity might play a role in its beneficial effects. To test this idea, the effects of iron and copper on both the neuroprotective and anti-inflammatory activities of fisetin were examined. It is shown that while fisetin can reduce the potentiation of cell death by iron and copper in response to treatments that lower glutathione levels, it is much less effective when the metals are combined with other inducers of oxidative stress. In addition, iron but not copper reduces the anti-inflammatory effects of fisetin in a dose-dependent manner. These effects correlate with the ability of iron but not copper to block the induction of the antioxidant transcription factor, Nrf2, by fisetin. In contrast, although the flavanone sterubin also binds iron, the metal has no effect on sterubin’s ability to induce Nrf2 or protect cells from toxic or pro-inflammatory insults. Together, these results suggest that while iron and copper binding could contribute to the beneficial effects of neuroprotective compounds in the context of neurodegenerative diseases, the consequences of this binding need to be fully examined for each compound.

## 1. Introduction

My laboratory has shown that the flavonoid fisetin (3,7,3′,4′ tetrahydroxyflavone) has both neuroprotective and cognition-enhancing activities [1]. Fisetin was first identified in a screen for compounds that could protect nerve cells from a form of oxidative stress-induced nerve cell death called oxytosis or ferroptosis [2]. Not only does fisetin have direct antioxidant activity but it can also maintain the levels of the major intracellular antioxidant, glutathione (GSH), in the presence of oxidative stress by inducing the transcription factors, Nrf2 and ATF4 [3]. Importantly, administration of fisetin via the diet prevented the loss of cognitive function in mouse models of both familial (APPswe/PS1dE9 (huAPP/PS1) double transgenic AD mice) [4] and sporadic (rapidly aging SAMP8 mice) [5] Alzheimer’s disease (AD). In addition, fisetin has shown efficacy in preclinical models of Parkinson’s disease, Huntington’s disease and amyotrophic lateral sclerosis as well as multiple models of stroke [forreview1]. Importantly, fisetin was found to have robust anti-inflammatory activity both in vitro [6,7,8] and in vivo [9]. Together, these observations suggest that fisetin has multiple properties that might make it useful for the treatment of age-related neurological diseases.

With aging there is an increase in both iron and copper in the brain and these levels are further increased in AD [10,11,12,13,14,15,16] suggesting that they might play a role in the cognitive dysfunction that is the primary characteristic of the disease. For instance, a correlation was found between accelerated cognitive decline and high copper intake [17]. Analysis of the brains of AD patients with magnetic resonance imaging (MRI) found that damage to the hippocampus, the region of the brain that plays a key role in memory, is associated with iron accumulation [18,19]. Moreover, increases in brain iron levels as determined by MRI were associated with a decrease in cognitive function [19,20]. Importantly, the iron accumulation occurs only in the parts of the brain that are involved in AD [18]. A more recent study which investigated the association between post-mortem iron levels with the clinical and pathological diagnosis of AD found a strong correlation between high levels of iron in the inferior temporal cortex and cognitive decline while no increase in iron was seen in the cerebellum, a part of the brain not affected by AD [21].

Not only can iron and copper accumulation contribute to increases in oxidative stress and the potentiation of nerve cell death by oxytosis/ferroptosis [22] but recent studies suggest they may also may play roles in cellular senescence, a pro-inflammatory cell fate associated with age-related diseases, including AD [23,24]. Furthermore, iron and copper chelators have shown beneficial effects in animal models of AD [11,25,26] as well as some limited positive results in human AD patients [24,27]. However, these human results are tempered by the neurotoxicity associated with one of these compounds, cliquinol [28,29], as well as questions about its efficacy [30]. Indeed, the cliquinol clinical trials were stopped due to severe neurotoxic effects due to oxidative damage [29].

Metal chelation has been implicated in the beneficial effects of a number of different flavonoids including fisetin [31]. Indeed, fisetin has been reported to bind both iron [31,32,33,34] and copper [33,35]. Therefore, in this study it was asked if at least some of the neuroprotective and/or anti-inflammatory properties of fisetin could be due to its ability to modulate the effects of iron and/or copper on cells.

## 2. Materials and Methods

### 2.1. Materials

The source of FeCl_2_, CuCl_2_ and all other chemicals and reagents was Sigma-Aldrich (St. Louis, MO, USA) unless another company is listed.

### 2.2. Oxytosis Assay

The HT22 mouse hippocampal nerve cells are grown in high-glucose Dulbecco’s modified Eagle’s medium (DMEM) (Invitrogen, Carlsbad, CA, USA) which is supplemented with 10% fetal calf serum (FCS) (Invitrogen). The cells were plated in 96-well plates at 5 × 10^3^ cells/well. After 24 h of culture, the medium was exchanged with fresh medium and glutamate [36] and fisetin alone (5 µM) or 5 µM fisetin (Indofine, Hillsborough, NJ, USA) and different amounts of FeCl_2_ or CuCl_2_ (0.5–10 µM) were added to the cells. In some cases, the oxidants H_2_O_2_ or t-butylperoxide or the glutathione peroxidase 4 inhibitor RSL3 (Cayman Chemical, Ann Arbor, MI, USA) were used instead of glutamate to induce cell death. After 24 h of treatment, viability was measured by the 3-(4, 5-dimethylthiazolyl-2)-2,5-diphenyltetrazolium bromide (MTT) assay as previously described [36,37]. The results of the MTT assay were confirmed by visual observation of the wells. To test for the effects of the metals on cell survival and proliferation, controls with the metals alone were included.

### 2.3. Inflammation Assay

In this assay, mouse BV2 microglial cells grown in low glucose DMEM supplemented with 10% FCS were plated at 5 × 10^5^ cells in 35 mm tissue culture dishes. After growth overnight, the cells were treated with 25 µg/mL bacterial lipopolysaccharide (LPS) alone or in the presence of fisetin alone (5 µM) or 5 µM fisetin and different amounts of FeCl_2_ or CuCl_2_ (0.5–10 µM). After 24 h the medium was removed, spun briefly to remove floating cells and 100 µL assayed for nitrite as described previously [38]. ELISAs (R&D Systems, Minneapolis, MN, USA) were used to assess the levels of IL-6 and TNFα in the supernatants following the manufacturer’s instructions.

### 2.4. Enzymatic Measurement of Total Glutathione (tGSH)

For measurement of total GSH, 3 × 10^5^ HT22 cells were plated in 60 mm dishes. After 24 h of culture, the medium was exchanged with fresh medium and the indicated concentrations of glutamate, 5 µM fisetin alone or 5 µM fisetin and different amounts of FeCl_2_ or CuCl_2_ (0.5–10 µM) were added to the cells. The cells were treated for 24 h or as indicated in the figure legends and then prepared and analyzed for GSH as described previously [3]. The GSH content was normalized to total protein in the solubilized acid-precipitated pellet using the bicinchoninic acid (BCA) assay (Pierce, Rockford, IL) [3]. To compare the effects of the treatments on cytoplasmic versus mitochondrial GSH levels, 9 × 10^5^ HT22 cells were plated in 100 mm dishes and treated exactly as the cells in 60 mm dishes. At the end of the treatment, the cells were separated into cytoplasmic, nuclear and mitochondrial fractions by differential centrifugation [39] and then the cytoplasmic and mitochondrial fractions were assaysed for GSH as described above for the total cell extract.

### 2.5. Reactive Oxygen Species (ROS) Measurement

HT22 cells were seeded onto 96-well black walled microtiter plates at a density of 1 × 10^4^ cells per well. The next day, the cells were treated with 10 µM fisetin alone or in the presence of FeCl_2_ or CuCl_2_ and/or in the presence of 5 mM glutamate for 7–8 h or 250 nM RSL3 for 4 h. The medium was then replaced with 100 µL loading medium (phenol red-free Hank’s balanced salt solution containing 10 µM CM-H_2_DCFDA (C6827, Invitrogen)). After 30 min, the fluorescence (λ excitation = 495 nm, λ emission = 525 nM) was determined using a Molecular Devices microplate reader. Each treatment was done in sextuplicate. DCFDA fluorescence was normalized to control cells not exposed to metals, glutamate or fisetin.

### 2.6. Determination of Intracellular Iron and Copper Binding 

HT22 cells were seeded onto 96-well black walled microtiter plates at a density of 1 × 10^4^ cells per well. The next day, the cells were treated with fisetin alone or in the presence of FeCl_2_ or CuCl_2_ for 3 h. The medium was then replaced with 100 µL loading medium (phenol red-free Hank’s balanced salt solution containing either 2 µM Phen Green FL diacetate (copper; P6763, Invitrogen) or 2 µM Phen Green SK diacetate (iron, P14313, Invitrogen). After 30 min, the fluorescence (λ excitation = 490 nm, λ emission = 520 nM for Phen Green FL ανδ λ excitation = 507 nm, λ emission = 532 nM for Phen Green SK) was determined using a Molecular Devices microplate reader. Each treatment was done in sextuplicate. Fluorescence was normalized to control cells not exposed to the metals.

### 2.7. Determination of the Trolox Equivalent Activity Concentration (TEAC)

TEAC values for fisetin were determined as described previously [40].

### 2.8. Protein Preparation and Western Blotting

For the analysis by Western blotting, 24 h prior to the indicated treatments, 3 × 10^5^ HT22 cells were plated on 60 mm dishes. After the treatments, nuclear extracts were prepared as described [3]. The protein concentrations of the extracts were determined using the BCA assay and equal amounts were solubilized in sample buffer as described [3].

Equal amounts of protein (10–20 µg per lane) were used for SDS-PAGE. The proteins were separated on 10% Criterion XT Precast Bis-Tris Gels (Biorad, Hercules, CA, USA) and transferred to nitrocellulse using a semi-dry transfer apparatus (Transblot, Biorad). The blots were stained with Ponceau S to ascertain the quality of protein measurement, electrophoresis and transfer efficiency. The nitrocellulse membranes were blocked for 1 h at room temperature with 5% non-fat milk in TBS-T (20 mM Tris buffer pH 7.5, 0.5 M NaCl, 0.1% Tween 20) and then at 4 °C overnight in the primary antibody diluted in 5% BSA in TBS/0.05% Tween 20. The primary antibodies used were: rabbit anti-Nrf2 (#sc-13032, 1/500) and rabbit anti-ATF4 (#sc-200, 1/500) from Santa Cruz Biotechnology (Dallas, TX, USA) and HRP-conjugated rabbit anti-actin (#5125, 1/20,000) from Cell Signaling (Danvers, MA, USA). The anti-ATF4 and anti-Nrf2 blots were rinsed with TBS/0.05% Tween 20 and incubated for 1 h at room temperature in horseradish peroxidase-goat anti-rabbit (Biorad, Hercules, CA) diluted 1/5000 in 5% nonfat milk in TBS/0.1% Tween 20. The immunoblots were developed with the Super Signal reagent (Pierce, Rockford, IL). For both antibodies, the same membrane was re-probed with the HRP anti-actin antibody. Autoradiographs were scanned using a Biorad GS800 scanner and the band density was determined using the manufacturer’s software. Each Western blot was performed a minimum of three times with independent protein samples.

### 2.9. Statistical Analysis

All of the experiments were performed at least in triplicate and were repeated a minimum of three times. The statistical analysis of the results was done using GraphPad Prism 7 and either the t-test or analysis of variance (ANOVA) test and Tukey’s post test for individual group means comparisons as appropriate. The results were analyzed for statistically significant differences with *p* < 0.05 considered significant.

## 3. Results

To examine the effects of iron and copper on fisetin-mediated neuroprotection, I used the oxytosis assay in conjunction with the HT22 hippocampal nerve cell line. This assay tests the ability of compounds to rescue cells from oxidative stress-induced regulated cell death initiated by glutathione (GSH) depletion [41]. Because of the mechanistic association of the oxytosis assay with aging and age-associated neurodegenerative diseases such as AD, it is an excellent model for studying pathways involved in nerve cell damage and death in these diseases [42,43,44]. Importantly, recent studies indicate that iron [22,45] and copper [22] can both exacerbate the cell death initiated by GSH depletion.

### 3.1. Addition of Fisetin Shifts the Dose Response Curve for Iron and Copper Potentiation to Higher Concentrations

**Figure 1:**
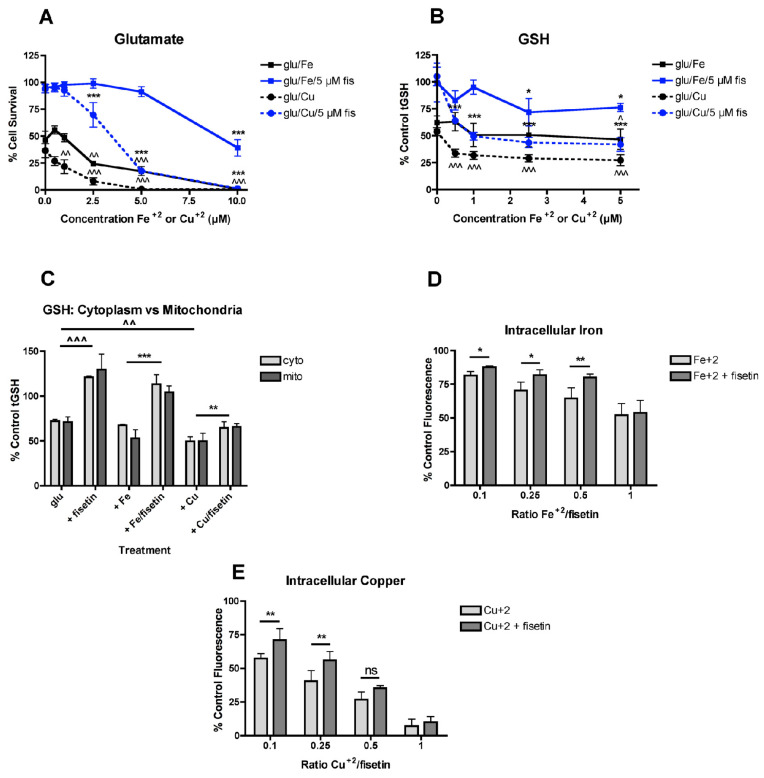
FeCl_2_ and CuCl_2_ modulate the effects of fisetin on survival and GSH. (**A**) Fisetin reduces the potentiation of glutamate toxicity by FeCl_2_ and CuCl_2_. HT22 cells were treated with 2.5 mM glutamate in the presence of 5 µM fisetin and/or FeCl_2_ or CuCl_2_ at the indicated concentrations. Cell survival was measured after 24 h with the MTT assay. The experiments were done in quadruplicate and the results are the average of 5–6 independent experiments. (**B**) Fisetin reduces the potentiation of total GSH loss by FeCl_2_ and CuCl_2_. HT22 cells were treated with 2.5 mM glutamate in the presence of 5 µM fisetin and/or FeCl_2_ or CuCl_2_ at the indicated concentrations. Cells were harvested after 24 h and total GSH was measured. The results are the average of 5-6 independent experiments. * indicates *p* < 0.05 and *** indicates *p* < 0.001 relative to fisetin + glutamate alone and ^ indicates *p* < 0.05, ^^ indicates *p* < 0.01 and ^^^ indicates *p* < 0.001 relative to glutamate alone. (**C**) Fisetin similarly reduces the potentiation of total GSH loss by FeCl_2_ and CuCl_2_ in both cytoplasm and mitochondria. HT22 cells were treated with 2.5 mM glutamate in the presence of 10 µM fisetin and/or 5 µM FeCl_2_ or CuCl_2_ (0.5:1, metal:fisetin). Cells were harvested after 24 h, separated into cytoplasmic and mitochondrial fractions and total GSH was measured. The results are the average of 4–6 independent experiments. ** indicates *p* < 0.01 and *** indicates *p* < 0.001 relative to fisetin + glutamate alone. ^^ indicates *p* < 0.01 and ^^^ indicates *p* < 0.001 relative to glutamate alone. (**D**) Fisetin reduces free intracellular Fe^2+^ levels at Fe^2+^:fisetin ratios of 0.5:1 and below. Free intracellular Fe^2+^ was measured using the dye PhenGreen SK after 3 h of treatment with 10 µM fisetin and the indicated concentrations of FeCl_2_. (**E**) Fisetin reduces free intracellular Cu^2+^ levels at Cu^2+^:fisetin ratios of 0.25:1 and below. Free intracellular Cu^2+^ was measured using the dye PhenGreen FL after 3 h of treatment with 10 µM fisetin and the indicated concentrations of CuCl_2_. The experiments were done in sextuplicate and the results are the average of 4–6 independent experiments. * indicates *p* < 0.05 and ** indicates *p* < 0.01 relative to the metal alone.

Recently, it was shown that Fe^2+^ and Cu^2+^ can potentiate oxytosis in a dose-dependent manner [22] so it was asked if fisetin could reduce this potentiation and how that related to the metal:fisetin ratio. Fisetin was tested at 5 µM, a concentration that provides excellent protection [3]. For these experiments, the metals and fisetin were added separately alone or in the presence of glutamate and the effects on cell survival determined. While the addition of 2.5 µM Fe^2+^ significantly potentiated glutamate toxicity (Figure 1A), the presence of 5 µM fisetin shifted this dose to 10 µM Fe^2+^. Similarly, the dose response curve for the potentiation of toxicity by Cu^2+^ was also shifted significantly by 5 µM fisetin. However, fisetin was less effective at protecting against potentiation of oxytosis by Cu^2+^ than by Fe^2+^. Together, these results indicate that fisetin can modulate the potentiation of oxytosis by transition metals prompting further investigation into the mechanisms underlying this effect.

### 3.2. Fisetin Reduces the Effects of Iron and Copper on GSH Loss

Since GSH loss is a key step in oxytosis [41] and fisetin is able to reduce glutamate-induced loss of GSH [2,3,40], the effects of fisetin on Fe^2+^- or Cu^2+^-potentiated GSH loss in glutamate-treated cells were examined next. As shown in Figure 1B, when Fe^2+^ or Cu^2+^ and 5 µM fisetin were added separately in the presence of glutamate, fisetin was able to shift the dose response curve for GSH loss to higher concentrations of the metals. Similar to the results of the oxytosis assay, fisetin was less effective at protecting against potentiation of GSH loss by Cu^2+^ than by Fe^2+^. Similar results were obtained when the cells were fractionated and GSH levels were measured in both the cytoplasmic and mitochondrial fractions (Figure 1C) with fisetin less effective at preventing the potentiation of GSH loss by Cu^2+^ than by Fe^2+^.

### 3.3. Effects of Fisetin on the Intracellular Levels of Iron and Copper

In order to determine how these effects of fisetin on the potentiation of GSH loss and cell death by Fe^2+^ or Cu^2+^ correlated with the ability of fisetin to bind the metals within the HT22 cells, fluorescent dyes sensitive to the metals were used [46,47]. In both cases, the fluorescence of the dye is quenched by the free metal so if fisetin binds the metal, then the fluorescence should be increased relative to the level in the presence of the metal alone. As shown in Figure 1D, fisetin was able to reduce the fluorescence quenching by Fe^2+^ at Fe^2+^:fisetin ratios of 0.5:1 and below. Fisetin was less effective at reducing the fluorescence quenching by Cu^2+^ with no significant effects seen at a Cu^2+^:fisetin ratio higher than 0.25:1 (Figure 1E).

### 3.4. Iron but Not Copper Inhibits the Induction of Nrf2 by Fisetin

**Figure 2:**
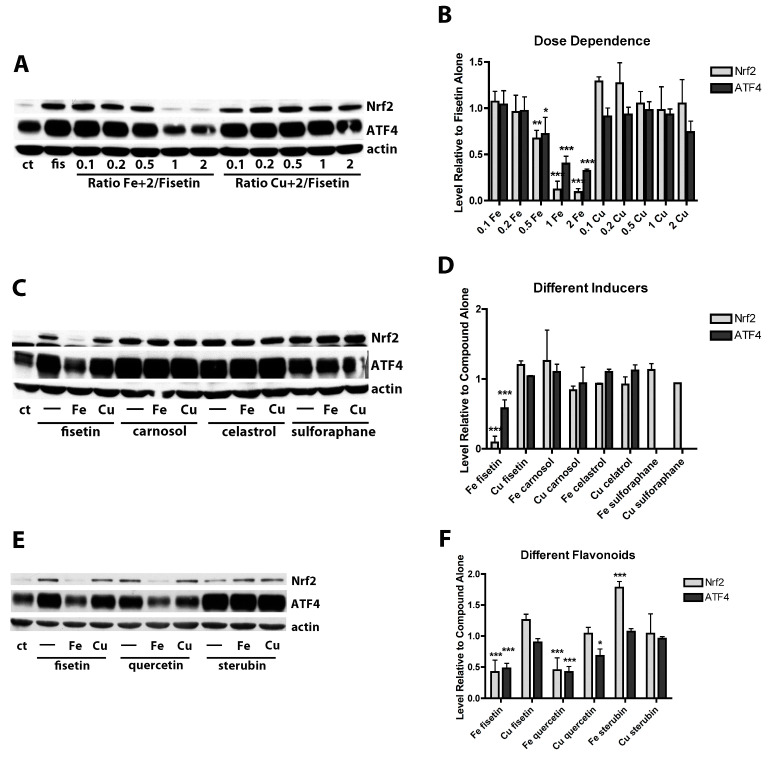
Effects of FeCl_2_ and CuCl_2_ on fisetin-induced increases in the transcription factors Nrf2 and ATF4. (**A**) Representative Western blots for Nrf2, ATF4 and actin of nuclear extracts from HT22 cells treated for 4 h with 5 µM fisetin either alone (fis) or in the presence of increasing concentrations of FeCl_2_ or CuCl_2_. (**B**) Quantitation of results from four independent experiments identical to (**A**). (**C**) Representative Western blots for Nrf2, ATF4 and actin of nuclear extracts from HT22 cells treated for 4 h with 5 µM fisetin, 1 µM carnosol, 1 µM celastrol or 5 µM sulforaphane either alone (-) or in the presence of a 2 fold molar excess of FeCl_2_ or CuCl_2_. (**D**) Quantitation of results from three independent experiments identical to (**C**). Since sulforaphane did not induce ATF4, no results for this are shown. (**E**) Representative Western blots for Nrf2, ATF4 and actin of nuclear extracts from HT22 cells treated for 4 h with 5 µM fisetin, 5 µM quercetin or 5 µM sterubin either alone (-) or in the presence of 10 µM FeCl_2_ or CuCl_2_. (**F**) Quantitation of results from four independent experiments identical to (**E**). In (**B**,**D**), * indicates *p* < 0.05, ** indicates *p* < 0.01 and *** indicates *p* < 0.001 relative to fisetin alone. In (**F**), * indicates *p* < 0.05 and *** indicates *p* < 0.001 relative to compound alone.

Previously, we showed that fisetin maintained GSH levels in the presence of oxidative stress partly by increasing the levels of the transcription factors Nrf2 and ATF4 [3]. To determine if some of the effects of the metals on fisetin’s ability to maintain GSH levels in the presence of oxidative stress were related to inhibition of fisetin’s ability to induce Nrf2 and/or ATF4, nuclei were prepared from control cells and cells treated with 5 µM fisetin alone or in the presence of increasing concentrations of Fe^2+^ or Cu^2+^. Surprisingly, while Cu^2+^ had no significant effect on the ability of fisetin to increase Nrf2 or ATF4 levels at any of the concentrations tested, Fe^2+^ significantly reduced the levels of Nrf2 and ATF4 induced by fisetin at a 0.5:1 (Fe^2+^:fisetin) ratio and above (Figure 2A,B) although the effect on Nrf2 levels was greater than the effect on ATF4 levels (Figure 2B). However, neither Fe^2+^ nor Cu^2+^ had any effect on the ability of several other Nrf2 inducers including carnosol, celastrol and sulforaphane to induce Nrf2 or ATF4 (Figure 2C,D).

It was then asked if Fe^2+^ also interfered with Nrf2 or ATF4 induction by other flavonoids. Quercetin is a potent Nrf2 inducer [48], has also been shown to bind Fe^2+^ [49] and is structurally very closely related to fisetin with the only difference being an additional hydroxyl group at the 5 position. We recently showed that the flavanone sterubin is also a strong inducer of Nrf2 and binds Fe^2+^ [50]. Interestingly, similar to the results with fisetin, Fe^2+^ but not Cu^2+^ interfered with the ability of quercetin to induce Nrf2 and ATF4 but neither metal had any effect on the induction of Nrf2 or ATF4 by sterubin (Figure 2E,F).

### 3.5. Effects of Iron and Copper on the Antioxidant Activity of Fisetin

**Figure 3:**
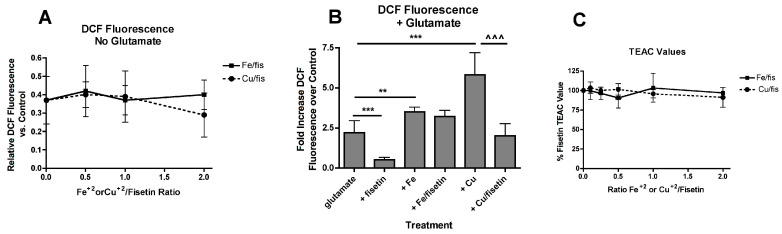
Effects of FeCl_2_ and CuCl_2_ on fisetin-mediated reductions in ROS levels. (**A**) HT22 cells were treated with 10 µM fisetin and increasing concentrations of FeCl_2_ or CuCl_2_. After 7 hr, ROS levels were determined using CM-H_2_DCFDA as described in Materials and Methods. The treatments were done in sextuplicate and the results are the average of 4 independent experiments. (**B**) Cells were treated with 5 mM glutamate alone or in the presence of 10 µM fisetin and/or 10 µM of FeCl_2_ or CuCl_2_. After 7 h, ROS levels were determined using CM-H_2_DCFDA as described in Materials and Methods. The treatments were done in sextuplicate and the results are the average of 4 independent experiments. ** *p* < 0.01, *** *p* < 0.001 relative to glutamate alone. ^^^ *p* < 0.001 relative to glutamate + CuCl_2_ alone. (**C**) TEAC values were determined for fisetin in the presence of increasing concentrations of FeCl_2_ or CuCl_2_ as described in Materials and Methods.

Recently, it was shown that Fe^2+^ and Cu^2+^ increase the levels of reactive oxygen species (ROS) in control cells as measured using the dye CM-H_2_DCFDA [22]. Increases in ROS also play a key role in the oxytosis pathway [41]. Fisetin was able to prevent the metal-induced increases in ROS in control cells (Figure 3A). However, while 5 µM fisetin was also very effective at reducing the increases in ROS production following glutamate treatment, the presence of 10 µM Fe^2+^ blocked that effect. Surprisingly, 10 µM Cu^2+^ interfered much less with the ability of fisetin to suppress glutamate-induced ROS (Figure 3B).

Fisetin has direct antioxidant activity which might play a role in its ability to both suppress ROS production and maintain GSH levels and this activity might be affected differentially by the metals. This direct antioxidant activity can be assessed using the Trolox Equivalent Activity Assay (TEAC) [40] where fisetin has a reported TEAC value of ~3 [2]. This value did not change significantly in the presence of increasing concentrations of Fe^2+^ or Cu^2+^ (Figure 3C) consistent with the lack of effect of Fe^2+^ or Cu^2+^ on basal ROS levels (Figure 3A). These results are also consistent with the lack of correlation between iron binding and radical scavenging for a number of flavonoids, including fisetin, seen in an earlier study [31]. Thus, direct interference of Fe^2+^ or Cu^2+^ with the direct antioxidant activity of fisetin cannot explain the differential effects of the metals on the ability of fisetin to suppress glutamate-induced ROS production.

### 3.6. Iron More than Copper Dose-Dependently Reduces the Protection by Fisetin against Multiple Stresses

**Figure 4:**
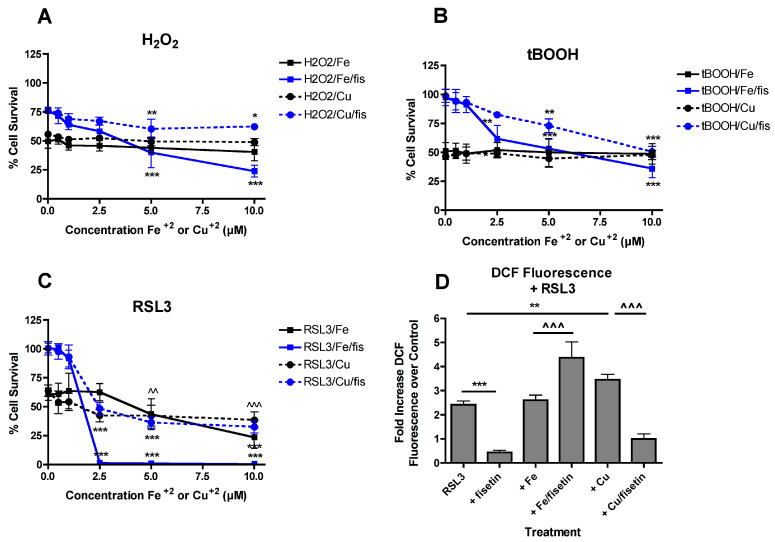
FeCl_2_ and CuCl_2_ modulate the effects of fisetin on protection from multiple toxicities. FeCl_2_ and CuCl_2_ reduce fisetin-mediated protection against hydrogen peroxide (H_2_O_2_) (**A**) or t-butyl peroxide (tBOOH) (**B**) toxicity. HT22 cells were treated with 0.5 mM H_2_O_2_ or 2.5 µM tBOOH in the presence of 10 µM fisetin and/or FeCl_2_ or CuCl_2_ at the indicated concentrations. Cell survival was measured after 24 h with the MTT assay. The experiments were done in quadruplicate and the results are the average of 4 independent experiments. (**C**) FeCl_2_ and CuCl_2_ differentially affect fisetin-mediated protection against RSL3 toxicity. HT22 cells were treated with 100 nM RSL3 in the presence of 5 µM fisetin and/or FeCl_2_ or CuCl_2_ at the indicated concentrations. Cell survival was measured after 24 h with the MTT assay. The experiments were done in quadruplicate and the results are the average of 5 independent experiments. (**A**–**C**), * indicates *p* < 0.05, ** indicates *p* < 0.01 and *** indicates *p* < 0.001 relative to fisetin + toxin alone. (**C**), ^^ indicates *p* < 0.01 and ^^^ indicates *p* < 0.001 relative to RSL3 alone for both FeCl_2_ and CuCl_2_. (**D**) Cells were treated with 250 nM RSL3 alone or in the presence of 10 µM fisetin and 5 µM FeCl_2_ or CuCl_2_. After 4 h, ROS levels were determined using CM-H_2_DCFDA as described in Materials and Methods. The treatments were done in sextuplicate and the results are the average of 4 independent experiments. ** *p* < 0.01, *** *p* < 0.001 relative to RSL3 alone. ^^^ *p* < 0.001 relative to RSL3 + FeCl_2_ or CuCl_2_ alone.

Given the distinct effects of Fe^2+^ or Cu^2+^ on the ability of fisetin to prevent glutamate-induced ROS production (Figure 3), it was next asked whether Fe^2+^ and/or Cu^2+^ altered fisetin-mediated protection against oxidative stresses not associated with GSH depletion. Recently, it was shown that neither Fe^2+^ nor Cu^2+^ potentiated the toxicity of t-butyl peroxide or hydrogen peroxide [22]. Here, the effects of the metals on fisetin-mediated protection against t-butyl peroxide and/or hydrogen peroxide toxicity were examined. As shown in Figure 4A,B, Fe^2+^ reduced protection by 10 µM fisetin against both insults at concentrations of 5 µM and above while Cu^2+^ had a much more modest effect.

RSL3 is an inhibitor of glutathione peroxidase 4 (GPx4) [51] which induces a form of cell death called ferroptosis which is very similar, if not identical, to the form of cell death induced by treatment with glutamate [42]. However, its toxicity does not involve GSH depletion. As shown in Figure 4C, both Fe^2+^ and Cu^2+^ alone potentiated RSL3 toxicity consistent with the effects of both metals on glutamate toxicity. Surprisingly, 5 µM fisetin enhanced the potentiation of RSL3 toxicity by Fe^2+^ at concentrations of 2.5 µM and above but had little or no effect on the potentiation of RSL3 toxicity by Cu^2+^ at the same ratios.

Since fisetin did not prevent the potentiation of glutamate-induced ROS production by Fe^2+^ (Figure 3) and ROS are required for the initiation of lipid peroxidation which drives RSL3-mediated cell death, it was asked if a similar response was seen with RSL3 treatment. HT22 cells were treated for 4 h with RSL3 alone or in the presence of fisetin and/or the metals. As shown in Figure 4D, fisetin completely blocked ROS production by RSL3. While Cu^2+^ enhanced RSL3-mediated ROS production, this was also completely prevented by fisetin. In contrast, while Fe^2+^ alone did not further increase RSL3-mediated ROS production, it was significantly increased in the presence of fisetin consistent with the potentiation of RSL3 toxicity by the combination of Fe^2+^ and fisetin.

### 3.7. Effects of Iron and Copper on the Anti-inflammatory Effects of Fisetin

**Figure 5:**
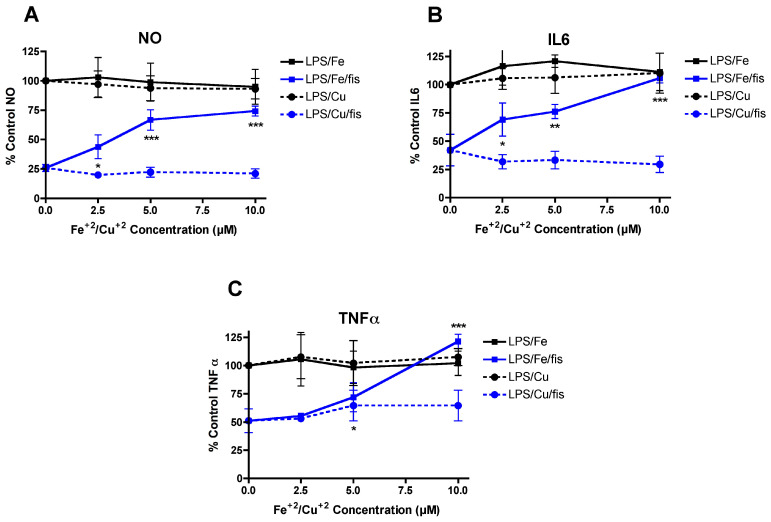
FeCl_2_ and CuCl_2_ differentially modulate the anti-inflammatory effects of fisetin. Dose dependent effects of FeCl_2_ and CuCl_2_ on the inhibition of NO and pro-inflammatory cytokine production by fisetin in LPS-treated BV-2 microglial cells. BV-2 cells were treated overnight with 25 ng/mL LPS in the presence of 5 µM fisetin alone or with the addition of the indicated concentrations of FeCl_2_ or CuCl_2_. Cell culture supernatants were cleared and assayed for NO by the Griess assay or pro-inflammatory cytokines using specific ELISAs. Results are presented as the percent (%) of the value obtained with LPS alone which was set at 100%. (**A**) NO, (**B**) IL6, (**C**) TNFα. The results represent the average of 3–4 independent experiments. * indicates *p* < 0.05, ** indicates *p* < 0.01 and *** indicates *p* < 0.001 relative to fisetin + LPS alone.

To determine if the interactions between Fe^2+^, Cu^2+^ and fisetin were specific to neuroprotection or were also seen with other activities of fisetin, we looked at the effects of Fe^2+^ and Cu^2+^ on the anti-inflammatory activity of fisetin using mouse BV-2 microglial cells. Multiple studies have shown that fisetin dose-dependently reduces the increase in both nitric oxide (NO) and pro-inflammatory cytokine production following LPS treatment of microglia [6,7,8,9]. The effects of Fe^2+^ and Cu^2+^ alone on LPS-induced stimulation of NO and cytokine production were examined first. As shown in Figure 5A–C, neither had any significant effect at the doses used (2.5–10 µM). However, while 5 µM fisetin alone reduced NO production by ~75% (Figure 5A), IL-6 production by ~60% (Figure 5B) and TNFα production by ~50% (Figure 5C), Fe^2+^ dose dependently reduced the anti-inflammatory effects of fisetin (Figure 5A–C). In contrast, Cu^2+^ had no effect or slightly enhanced the anti-inflammatory effects of fisetin (Figure 5A–C).

**Figure 6:**
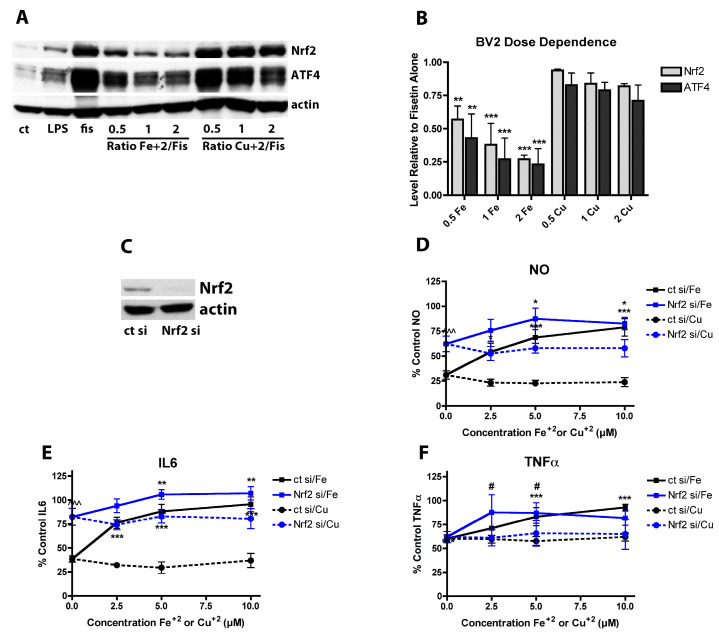
Nrf2 plays a key role in the anti-inflammatory effects of fisetin. (**A**) Representative Western blots for Nrf2, ATF4 and actin in nuclear extracts from BV-2 cells treated with 25 ng/mL LPS alone or in the presence of 5 µM fisetin ± the indicated concentrations of FeCl_2_ or CuCl_2_ for 4 h. (**B**) Quantitation of results from four independent experiments identical to (**A**). ** indicates *p* < 0.01 and *** indicates *p* < 0.001 relative to fisetin alone. (**C**) Representative Western blot of BV-2 cells treated for 48 h with control siRNA (ct si) or Nrf2 siRNA (Nrf2 si). (**D**–**F**) BV2 cells transfected with control siRNA (ct si) or Nrf2 siRNA (Nrf2 si) were treated overnight with 25 ng/mL LPS and 5 µM fisetin and/or the indicated concentrations of FeCl_2_ or CuCl_2_. Cell culture supernatants were cleared and assayed for NO by the Griess assay or pro-inflammatory cytokines using specific ELISAs. Results are presented as the percent (%) of the value obtained with LPS alone which was set at 100%. NO (**D**); IL6 (**E**) or TNFα (**F**). Results in (**D**–**F**) are the average of three independent experiments. * indicates *p* < 0.05, ** indicates *p* < 0.01 and *** indicates *p* < 0.001 relative to fisetin + LPS alone. # indicates *p* < 0.05 relative to Nrf2 siRNA with fisetin and LPS only.

Since it was found that induction of Nrf2 by fisetin was blocked by Fe^2+^ but not Cu^2+^ (Figure 2) and Nrf2 activates anti-inflammatory pathways [52], it was next asked whether inhibition of Nrf2 induction by Fe^2+^ contributed to the Fe^2+^-mediated decrease in the anti-inflammatory effects of fisetin. First, the effects of fisetin ± Fe^2+^ or Cu^2+^ on nuclear Nrf2 levels were examined. BV-2 cells treated for 24 h with 5 µM fisetin showed a large increase in nuclear Nrf2 levels and this was significantly reduced by Fe^2+^ but not Cu^2+^ (Figure 6A,B) at all doses of the metals that were tested. Next, Nrf2 was knocked down in BV-2 cells with siRNA (Figure 6C; average knockdown = 82 ± 9%) and the anti-inflammatory effects of fisetin on LPS-treated cells in the absence and presence of the metals was examined. BV-2 cells treated with control siRNA behaved almost identically to untreated cells with regard to the effects of fisetin on LPS-stimulated NO and cytokine production (Figure 6D–F). In contrast, in BV-2 cells treated with Nrf2 siRNA, the anti-inflammatory effects of fisetin were greatly diminished with regard to NO and IL-6 production but not TNFα production (Figure 6D–F). In the absence of Nrf2, Fe^2+^ only modestly further reduced the effects of fisetin on NO and IL-6 production. Knockdown of Nrf2 had a much lesser effect on the reduction of fisetin-induced TNFα production by Fe^2+^. ATF4 knockdown (not shown) did not blunt the effects of Fe^2+^ on the fisetin-mediated suppression of NO or IL-6 production. Together, these results suggest that the negative effects of Fe^2+^ on fisetin-induced Nrf2 upregulation could at least partly explain its inhibition of the anti-inflammatory actions of fisetin.

### 3.8. Fe^2+^ Has a Minimal Effect on the Neuroprotective and Anti-inflammatory Effects of Sterubin

**Figure 7:**
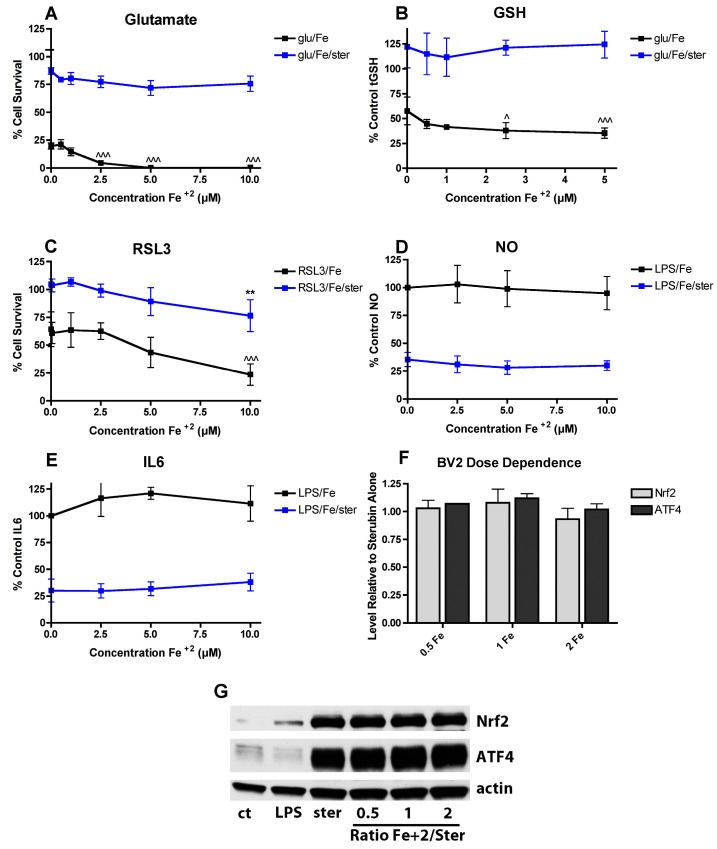
FeCl_2_ does not interfere with the activities of sterubin. (**A**) FeCl_2_ does not alter the protective effect of sterubin against glutamate toxicity. HT22 cells were treated with 2.5 mM glutamate in the presence of 5 µM sterubin and/or FeCl_2_ at the indicated concentrations. Cell survival was measured after 24 h with the MTT assay. The experiments were done in quadruplicate and the results are the average of 4 independent experiments. (**B**) FeCl_2_ does not affect the maintenance of GSH levels by sterubin. HT22 cells were treated with 2.5 mM glutamate in the presence of 5 µM sterubin and/or FeCl_2_ at the indicated concentrations. Cells were harvested after 24 h and total GSH was measured. The results are the average of 5–6 independent experiments. (**C**) FeCl_2_ only slightly impairs the protective effect of sterubin against RSL3 toxicity. HT22 cells were treated with 100 nM RSL3 in the presence of 5 µM sterubin and/or FeCl_2_ at the indicated concentrations. Cell survival was measured after 24 h with the MTT assay. The experiments were done in quadruplicate and the results are the average of 4 independent experiments. (**D**–**E**) FeCl_2_ does not reduce the anti-inflammatory effects of sterubin. BV-2 cells were treated overnight with 25 ng/mL LPS in the presence of 5 µM sterubin alone or with the addition of the indicated concentrations of FeCl_2_. Cell culture supernatants were cleared and assayed for NO by the Griess assay or IL-6 using a specific ELISA. Results are presented as the percent (%) of the value obtained with LPS alone which was set at 100%. ** indicates *p* < 0.01 relative to sterubin + RSL3 alone. ^ indicates *p* < 0.05 and ^^^ indicates *p* < 0.001 relative to insult alone. (**F**) Quantitation of results from four independent experiments identical to (**G**). (**G**) Representative Western blots for Nrf2, ATF4 and actin of nuclear extracts from BV-2 cells treated with 25 ng/mL LPS alone or in the presence of 5 µM sterubin ± the indicated concentrations of FeCl_2_ or CuCl_2_ for 4 h.

Since we recently showed that sterubin has neuroprotective and anti-inflammatory activity in the same assays as described above with fisetin [50] and here found that Fe^2+^ does not interfere with the ability of sterubin to induce Nrf2 or ATF4 (Figure 2E,F), it was next asked what effect Fe^2+^ has in these assays on the actions of sterubin. As shown in Figure 7, Fe^2+^ had no effect on the protective effect of 5 µM sterubin in the oxytosis assay (Figure 7A) nor did it interfere with the ability of 5 µM sterubin to maintain GSH levels in the presence of glutamate (Figure 7B). Similarly, Fe^2+^ did not reduce the protective effects of 5 µM sterubin against RSL3 toxicity (Figure 7C). Consistent with the results with the HT22 cells, Fe^2+^ did not alter Nrf2 induction by 5 µM sterubin in the BV2 cells (Figure 7D) and had no effect on the strong anti-inflammatory effects of 5 µM sterubin against NO and IL-6 production in the BV2 cells (Figure 7E,F). Effects on TNFα were not examined as sterubin has only a very modest effect on LPS-induced TNFα production [50]. Consistent with these results, Fe^2+^, did not interfere with the induction of Nrf2 by sterubin in the BV2 cells (Figure 7G).

## 4. Discussion

The major observation from these studies is that the binding of iron or copper by the flavonol fisetin modulates its effects on cells. Importantly, the consequences of this binding appear to depend on both the type of cell and the type of insult. For example, while copper binding can reduce the protective effects of fisetin against oyxtosis, it slightly potentiates the anti-inflammatory effects of fisetin. In contrast, while iron has a dramatic effect on the anti-inflammatory effects of fisetin, it has a lesser impact on the protective effects of fisetin against oxytosis.

These differences appear, at least in part, to be due to the other important observation from this study which is that iron but not copper inhibits the induction of Nrf2 by fisetin. This effect of iron is observed in both HT22 hippocampal nerve cells and in BV-2 microglial cells and is not seen with several other Nrf2 inducers suggesting that the effect is specific to the mechanism underlying the induction of Nrf2 by fisetin and structurally related flavonoids such as quercetin. The exact mechanism is currently unclear but it is not due to the destabilization of Nrf2. The effect of fisetin on the half-life of nuclear Nrf2 [3] is similar in the absence or presence of iron (not shown). In contrast, iron does not interfere with the ability of the flavanone sterubin to induce Nrf2 and sterubin remains effective in all of the assays, even in the presence of excess iron.

Another reason for the differences in the effects of iron and copper on the activity of fisetin in the different cell assays could be due to the distinct contributions of iron and copper to the potentiation of oxytosis. Recently, it was shown that copper is a more potent potentiator of oyxtosis than iron [22]. Copper but not iron has multiple detrimental effects in this toxicity assay including inhibition of cystine uptake, which in its reduced form (cysteine) is the rate limiting amino acid in GSH biosynthesis, and inhibition of glutamate cysteine ligase activity, the rate limiting enzyme in GSH biosynthesis. Thus, any excess copper not bound by fisetin could still act on these targets to promote cell death. In addition, it was found that in cells fisetin is less effective at binding copper than iron (Figure 1C,D) which would further exacerbate this difference.

In contrast, the more dramatic effects of iron as compared to copper on fisetin-mediated protection against hydrogen peroxide toxicity could be due to the much stronger ability of iron to oxidize fisetin as compared to copper [33]. Importantly, oxidized fisetin was previously shown to be a potent pro-oxidant [53]. This would be consistent with the inability of fisetin to prevent the potentiation of glutamate-induced ROS production by iron but not copper. The iron-dependent oxidation of fisetin to generate a pro-oxidant could also, at least partly, explain the enhancing effects of fisetin on the iron-mediated potentiation of RLS3 toxicity. Inhibition of GPx4 by RSL3 leads to a large increase in lipid peroxidation [51] and since lipid peroxidation requires an initiator ROS [54], it is possible that oxidized fisetin can play this role. Indeed, while fisetin alone was very effective at preventing RSL3-induced ROS production, the combination of fisetin and iron enhanced ROS production by RSL3 while that of fisetin and copper did not.

A number of studies have looked at the binding of iron by fisetin [31,32,33,34]. Dimitric Markovic [34] et al. found that at neutral or alkaline pH, the chelating group was the catechol in the B ring and that fisetin bound iron in a 1:1 ratio. Several studies have also looked at copper binding by fisetin [33,35]. At neutral pH, fisetin also binds copper in a 1:1 ratio and, as with iron, the main chelating group is the catechol moiety in the B ring [35]. However, studies on other flavonols [55] found that the catechol group was associated with weak copper chelation activity. These differences in iron and copper binding by the catechol group could play a role in the distinct effects of the metals on the different activities of fisetin. However, while these test tube studies showed that fisetin bound iron and copper equally well, this does not seem to be the case is cells where fisetin is somewhat more effective at binding iron than copper and in neither case does there appear to be 1:1 binding of the metal with fisetin. This difference between the test tube and cells could be related to pH since at pH 7.5, both neutral and ionized forms of all of the ionizable hydroxyl groups of fisetin can co-exist [35].

Iron and copper are not toxic by themselves at the doses used in these studies but they promote the toxicity of various stresses, particularly those that induce GSH depletion [22]. GSH plays a key role in the maintainence of the cellular redox state [56]. Numerous studies have shown decreases in total GSH and/or reduced GSH levels in the brain with aging for reviews see [57,58]. Although many studies on GSH levels in the brain have been done on post-mortem tissue, more recent work has used live subjects in combination with double-edited ^1^H magnetic resonance spectroscopy (MRS). For example, when the GSH levels in the occipital cortex of healthy young (20 yr) and elderly (77 yr) human subjects were compared, they were found to be significantly decreased (~30%) in the elderly subjects [59]. Moreover, multiple studies in humans have demonstrated lower levels of GSH with age in plasma and/or red blood cells [57,60,61,62] and this age-dependent decrease is enhanced in mild cognitive impairment and AD [63]. AD patients also have lower levels of GSH in their brains for review see [64]. Indeed, a study that used ^1^H MRS to examine GSH levels in the hippocampus and frontal cortex of control, MCI and AD subjects showed a disease-dependent decrease in GSH levels in both brain regions that correlated with lower levels of cognitive function [65]. Importantly, the levels of GSH in the hippocampus could distinguish between healthy controls and MCI subjects while the levels of GSH in the cortex could distinguish between MCI and AD patients. Further analysis showed that the decreases in GSH were not secondary to tissue atrophy. Interestingly, two recent studies in mice that used knockdown of the catalytic subunit of glutamate cysteine ligase, the rate limiting enzyme for GSH biosynthesis in all neurons [66] or only forebrain neurons [67], to specifically reduce neuronal GSH levels, showed an enhanced, age-dependent development of cognitive deficits relative to control animals, supporting the idea that GSH loss contributes to cognitive impairments in aging and AD. Importantly, in the former study [66], the decrease in GSH levels in the hippocampus was modest but comparable to that seen in MCI and AD subjects [65]. Thus, age- and AD-dependent decreases in GSH levels may make the elderly particularly susceptible to damage associated with the age- and AD-dependent increases in the concentrations of iron and copper in the brain [14,25] and thereby initiate a feed forward cycle of cell damage and death. Thus, the results presented here suggest that compounds such as fisetin and sterubin that target both GSH loss and metal ion increases may provide a more effective approach to treating sporadic AD. This conclusion is supported by our recent study with fisetin in the SAMP8 mouse model of aging and sporadic AD [5].

However, as shown here, fisetin loses its effectiveness against both GSH loss and pro-inflammatory stimuli at equimolar and higher concentrations of iron. In contrast, copper only negatively impacts the effectiveness of fisetin against GSH loss and actually slightly enhances the anti-inflammatory activity of fisetin. Thus, the use of fisetin should be calibrated to the potential role of iron or copper in the specific condition being treated. Furthermore, the flavanone sterubin might provide an alternative to fisetin in conditions with an excess of iron since iron did not have an impact on the beneficial effects of sterubin in the various assays. Indeed, we recently showed that sterubin is effective at preserving memory in an acute model of AD [68].
